# The Progressive Increase of Food Waste in America and Its Environmental Impact

**DOI:** 10.1371/journal.pone.0007940

**Published:** 2009-11-25

**Authors:** Kevin D. Hall, Juen Guo, Michael Dore, Carson C. Chow

**Affiliations:** Laboratory of Biological Modeling, National Institute of Diabetes and Digestive and Kidney Diseases, Bethesda, Maryland, United States of America; Institute of Preventive Medicine, Denmark

## Abstract

Food waste contributes to excess consumption of freshwater and fossil fuels which, along with methane and CO_2_ emissions from decomposing food, impacts global climate change. Here, we calculate the energy content of nationwide food waste from the difference between the US food supply and the food consumed by the population. The latter was estimated using a validated mathematical model of metabolism relating body weight to the amount of food eaten. We found that US per capita food waste has progressively increased by ∼50% since 1974 reaching more than 1400 kcal per person per day or 150 trillion kcal per year. Food waste now accounts for more than one quarter of the total freshwater consumption and ∼300 million barrels of oil per year.

## Introduction

Recent spikes in food prices have led to increasing concern about global food shortages and the apparent need to increase agricultural production [1], [2]. Surprisingly little discussion has been devoted to the issue of food waste. Quantifying food waste at a national level is difficult because traditional methods rely on structured interviews, measurement of plate waste, direct examination of garbage, and application of inferential methods using waste factors measured in sample populations and applied across the food system [3]–[6]. In contrast, national agricultural production, utilization, and net external trade are tracked and codified in detailed food balance sheets published by the Food and Agriculture Organization of the United Nations (FAO) [7]. The food balance sheets provide a comprehensive assessment of the national food supply, including alcohol and beverages, adjusted for any change of food stocks over the reference period [8]. Since 1974, there has been a progressive increase in the per capita US food supply. Over the same period, there has also been an increase of body weight as manifested by the US obesity epidemic. We sought to estimate the energy content of food waste by comparing the US food supply data with the calculated food consumed by the US population.

Energy from ingested food supports basal metabolism and physical activities, both of which are functions of body weight. Surplus ingested energy is stored in the body and is reflected by a change of body weight. Because the average body weight of the US population has been increasing over the past 30 years, it is not immediately clear how much of the increased food supply was ingested by the population. Quantifying the food intake underlying an observed change of body weight requires knowing the energy cost of tissue deposition and the increased cost of physical activity and metabolic rate with weight gain. Here, we develop and validate a mathematical model of human energy expenditure that includes all of these factors and used the model to calculate the average increase of food intake underlying the observed increase of average adult body weight in the US since 1974 as measured by the US National Health and Nutrition Examination Survey (NHANES) [9].

## Results

**Figure 1:**
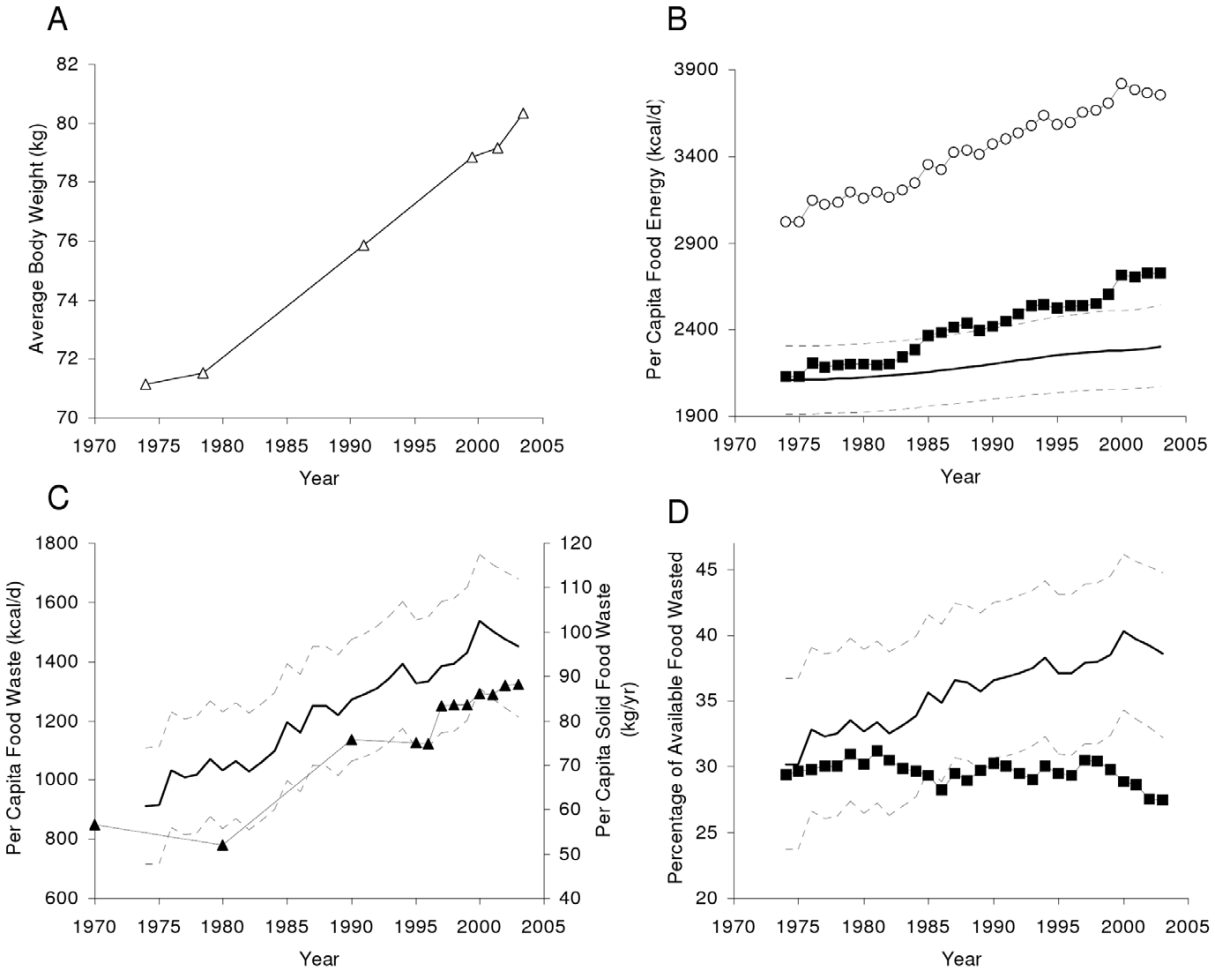
Food Supply, Intake, and Waste in America. (A) The average adult body weight (Δ) as measured by the National Health and Nutrition Examination Survey. (B). Per capita U.S. food availability unadjusted (○) and adjusted for wastage (▪) according to the United States Department of Agriculture (USDA). The solid curve represents the mathematical model prediction of average food intake change (dashed curves indicate±95% confidence intervals). (C) Energy content of per capita U.S. food waste predicted using our mathematical model (solid curve, left axis). The right axis plots the per capita annual mass of municipal solid food waste (▴). (D) Percentage of available food energy wasted as calculated by previous USDA estimates (▪) and predicted using our mathematical model (solid curve).

Figure 1A shows the increase of average body weight among US adults over the past 30 years (Δ). Assuming no change of physical activity, Figure 1B shows our model predicted average food intake (solid curve) and 95% confidence intervals (dashed curves) underlying the observed weight gain (see Methods for model details). Figure 1B also plots the US food supply data from the FAO food balance sheets (○)[7] and the US Department of Agriculture (USDA) food availability data adjusted for wastage (▪)[10] over the period 1974–2003. Figure 1C shows the progressive increase of per capita food waste in America (solid curve) calculated by subtracting the model predicted average food intake from the FAO per capita food supply data. In 1974 approximately 900 kcal per person per day was wasted whereas in 2003 Americans wasted ∼1400 kcal per person per day or ∼150 trillion kcal per year. Figure 1C shows that our estimate of the increasing energy content of US food waste is corroborated by the parallel increase of the per capita annual mass of municipal solid food waste (▴) calculated from data supplied by the US Environmental Protection Agency [11]. Municipal solid food waste accounts for ∼30% of the total wasted food energy assuming that solid food from the US diet has an energy density of 1.9 kcal/g [12]. Figure 1D shows that food waste has progressively increased from about 30% of the available food supply in 1974 to almost 40% in recent years (solid curve) whereas the USDA estimate of food waste (calculated by subtracting the USDA food availability data adjusted for spoilage and wastage from the FAO food supply data) was an approximately constant proportion of the total food supply (▪). While the USDA estimate of food waste was within 5% of our calculation in 1974, it was ∼25% too low in 2003.

## Discussion

The calculated progressive increase of food waste suggests that the US obesity epidemic has been the result of a “push effect” of increased food availability and marketing with Americans being unable to match their food intake with the increased supply of cheap, readily available food. Thus, addressing the oversupply of food energy in the US may help curb the obesity epidemic as well as decrease food waste, which has profound environmental consequences.

Assuming that agriculture utilizes about 70% of the freshwater supply [13], our calculations imply that more than one quarter of total freshwater use is accounted for by wasted food. Furthermore, given that the average farm requires 3 kcal of fossil fuel energy to produce 1 kcal of food (before accounting for energy requirements of food processing and transportation) [14], wasted food accounts for ∼300 million barrels of oil per year representing ∼4% of the total US oil consumption in 2003 [15]. In addition to this wasteful consumption of fossil fuels and their direct impact on climate change, food waste rotting in landfills produces substantial quantities of methane [16] – a gas with 25 fold more potent global warming potential than CO_2_ [17] which would have been the primary end product had the food been eaten and metabolized by humans.

Our food waste estimate resulted from subtracting the calculated average food energy intake from the food supply of the US population. Thus, there are two potential sources of error in our food waste estimate. First, the FAO food balance sheets were the source of our estimate of the US food supply [7]. The accuracy of food balance sheets has been questioned, especially for developing nations with a relatively high reliance on subsistence farming whose products rarely enter the marketplace and are therefore are difficult to account [8], [18]. While such issues are certainly less problematic for affluent nations like the US, there remain significant uncertainties regarding the absolute energy content of the food supply [8], [18]. However, our results rely primarily on the observed progressive increase of the food supply rather than its absolute level. Thus, unless the uncertainties of the US food supply data are systematically biased to progressively overestimate food supply at later dates, then our conclusions about the progressive increase of food waste remain valid.

**Figure 2:**
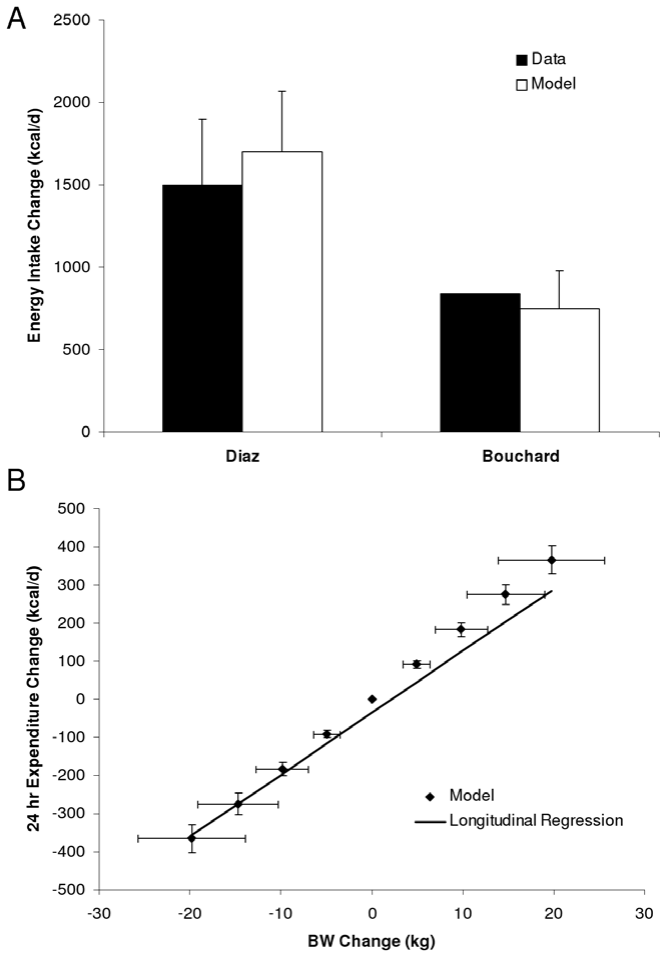
Mathematical model validation. (A) The experimentally imposed increases of food intake during controlled over-feeding experiments (black bars) along with model predicted values (white bars) calculated using the measured body weight changes. (B) Model predicted relationship between changes of 24 hour energy expenditure and body weight change after 3.6 years of over- and under-feeding (♦) along with the best-fit regression line determined from longitudinal measurements in a cohort of Pima Indians followed for the same average time interval. (mean±SD).

The second source of error in our calculation of food waste results from our mathematical model estimates of average food intake. The fact that average body weight of the US population has increased in parallel with the increasing food supply raises the question of how much of the additional available food was actually ingested by the population. Without an accurate mathematical model of human metabolism, we could not determine how increasing food intake quantitatively translates into a change of body weight. Figure 2A demonstrates that our model accurately calculated the energy intake changes underlying the observed weight gain in two controlled over-feeding experiments [19], [20] and Figure 2B shows that our model accurately predicted the relationship between weight change and energy expenditure in longitudinal data from a cohort of Pima Indians after a 3.6 year follow-up [21]. Compared to the 30 year time course of the NHANES data, we acknowledge that our model validation results are somewhat limited. Nevertheless, our model includes all of the main contributors to how food intake impacts body weight and we tested the robustness of our conclusions to uncertainties of the assumed parameter values by Monte Carlo sampling over parameter sets (see Methods) to generate the 95% confidence intervals (dashed curves in Figure 1). Furthermore, our estimate of a ∼50% increase of per capita food waste over the past 30 years is paralleled by a similar increase of per capita municipal solid food waste as depicted in Figure 1C thereby providing independent corroboration of our findings.

Our estimates of food waste likely represent lower bounds since we did not simulate the effects of a progressive decrease of physical activity that may have contributed to the US obesity epidemic [22]. However, some investigators contend that physical activity has not declined in the past few decades [23] which is in accordance with our model assumption. We have also not corrected the per capita adult food availability given that children consume less food than adults on an absolute basis. Accounting for both of these factors would increase the calculated food waste and therefore our estimates are conservative.

Our calculation of food wasted by the US population does not rely on direct measurements of waste produced by small groups that often know they are being investigated [6] nor individual assessments of food intake which are known to significantly underestimate actual food consumption [24]. Furthermore, inferential methods are prone to cumulative errors when using assumed food waste factors applied to various stages along the food system [3]–[5]. Previous estimates of food waste using these traditional methods have typically concluded that about one third of food mass is wasted [4], [5]. The USDA estimate that 27% of food is wasted is acknowledged to be an underestimate [4]. Therefore, the USDA food availability data is known to overstate the amount of food that people actually ingest [25]. Our results imply that the assumption of a roughly constant proportion of food waste calculated by the USDA has become progressively worse over time.

The principle of energy conservation implies that the energy content of food is closely related to the energy requirements for agricultural production as well as the methane and CO_2_ emissions produced by decomposition of wasted food. Thus, the energy content of wasted food may be a more important environmental index than the mass of wasted food as determined by more traditional methods. Nevertheless, traditional methods of measuring food waste provide important information about the types of foods wasted and the relative contribution of waste along various points of the supply chain from farm to fork. Because our methodology calculates food intake by the population and tracks food energy and not food types, we cannot address such issues. Nevertheless, the progressive deviation of our calculated wasted food energy compared with the USDA adjustment for wastage suggests that traditional methods are increasingly missing large quantities of food waste in America.

## Methods

The basis of our mathematical model is the energy balance equation where the rate of change in stored body energy is given by the difference between the metabolizable energy intake rate *I* and the energy expenditure rate *E*

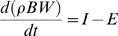
(1)where *BW* is the body weight, and *ρ* is the energy density of the change in the body weight. We can express the energy expenditure rate as
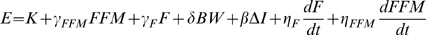
(2)where K is a constant, *γ_FFM_* = 22 kcal/kg/day and *γ_F_* = 3.2 kcal/kg/day are the regression coefficients relating resting metabolic rate versus fat-free mass (FFM) and fat mass (F), respectively [26]. Physical activity energy expenditure is proportional to body weight for most activities [27] and *δ* represents the level of physical activity. The parameter *β* accounts for the adaptation of energy expenditure during a diet perturbation Δ*I* and 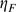 is 180 kcal/kg and 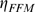 is 230 kcal/kg account for the biochemical cost of tissue deposition [28], [29] assuming that the change of FFM is primarily accounted for by body protein and its associated water [30]. We note that *FFM*, *F*, *I*, *BW*, *T* and *δ* are all functions of time.

Consider a population where each individual's weight change obeys equation (1) with their own individual intake and expenditure functions. We take a sample sum over (1) to obtain
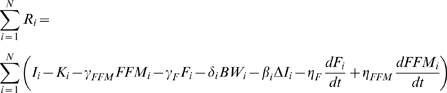
(3)where each subject is indexed by *i*, N is the number of subjects in the population, and 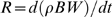 is the rate of change of energy stored in the body. Dividing both sides of equation (3) by N, gives us the sample mean of the population for all terms of the energy balance equation (1):
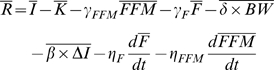
(4)


Since *FFM* = *BW*–*F*, we can rewrite equation (4) as
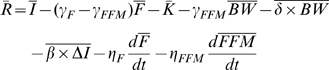
(5)


For the first NHANES phase from 1971–1974, we assumed that people were approximately weight-stable corresponding to a state of energy balance. Using the fact that 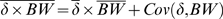 and 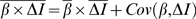, energy balance implies that the following equation must hold when the system is in an initial state of energy balance such that 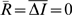:
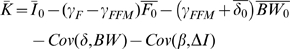
(6)


Therefore, assuming that the covariance of physical activity and body weight and the covariance of the parameter *β* with changes of food intake are approximately constant, substitution of equation 6 into equation 5 gives:
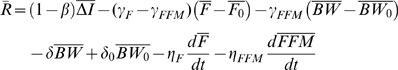
(7)where the average value of the parameter *β* = 0.24 was determined using under-feeding studies [29].

To estimate the rate of change of stored energy we consider fat and fat-free mass changes separately:
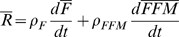
(8)where 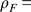 = 9400 kcal/kg and 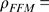 = 1800 kcal/kg are the energy densities for changes in fat and fat-free masses, respectively [30]. The relative change of *FFM* and *F* can be described by the Forbes theory of body composition change:
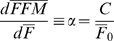
(9)where *C* = 10.4 kg is a parameter describing how body composition changes as a function of the initial body fat mass, *F*_0_ [31]. To calculate the value of the parameter α we required an estimate of the initial average body fat mass which was not directly measured in NHANES. We therefore estimated initial body fat mass from the body mass index (BMI) via the equations published by Jackson et al. [32] for men and women:
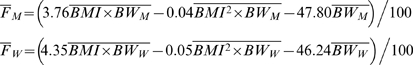
(10)where the mean values are taken over the men and women populations respectively. The population mean for the fat mass is then given by a weighted average of that of the men and women, 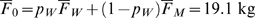, where 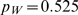 is the proportion of women in the NHANES population. This initial average fat mass is then used in equation (9) to calculate α = 0.54. 

Equation 9 implies that:
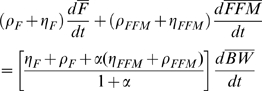
(11)and
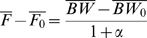
(12)


Therefore, substituting equations 11 and 12 into equation 7 gives the change of energy intake underlying the observed increase of average body weight:
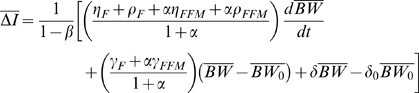
(13)


Using the nominal parameter values and assuming no change of physical activity, equation 13 can be represented as:
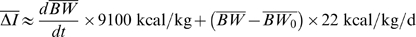
(13)


The first term of equation 14 evaluates to <10 kcal/d for the NHANES data since the rate of change of average body weight was only ∼9.5×10^−4^ kg/d. The second term evaluates to ∼220 kcal/d for the NHANES data since the change of average body weight was ∼10 kg between 1974 and 2003.

Our mathematical model was previously demonstrated to give accurate results in situations of underfeeding and body weight loss [29]. In the context of weight gain, we validated our model by predicting the changes of energy intake in the controlled feeding experiments of Diaz et al. [20] and Bouchard et al. [19] who overfed subjects by 1500±400 kcal/d and 840 kcal/d for 42 and 100 days, respectively. Figure 2A shows that using the observed weight changes, our model predicted that energy intake was increased by 1700±370 kcal/d and 750±230 kcal/d for the Diaz and Bouchard studies, respectively, which corresponds well with their actual intakes.

While these results give us some confidence in the validity of our model in response to weight gain, we note that the controlled over-feeding experiments were conducted in a small number subjects over a relatively short period of time. We could only find one study that measured longitudinal changes of energy expenditure with weight gain over extended time periods [21]. In that study, Weyer et al. investigated a cohort of Pima Indian subjects with an average 3.6 year follow-up and Figure 2B plots the best-fit regression line to the measured changes of energy expenditure (via indirect calorimetry) versus weight change [21]. Figure 2B also shows our model predictions (♦) of energy expenditure change as a function of body weight change in response to 3.6 years of over- and under-feeding to various degrees. While the model results correspond well with the regression line fit to the indirect calorimetry data, it is apparent that the model predicts a slightly greater slope than was indicated in the best-fit regression line. We hypothesize that the discrepancy is due to the limited physical activity of the study subjects during the measurement period inside the indirect calorimetry chamber. Since physical activity energy expenditure is proportional to body weight, decreased physical activity would result in a decreased slope of the relationship between energy expenditure versus weight change.

To calculate the absolute level of energy intake corresponding to the NHANES data, we assumed that the average initial energy intake was 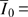 = 2100 kcal/d calculated using the energy requirement equations of the Institute of Medicine of the National Academies [33] for a sedentary population corresponding to the average demographics of the initial adult NHANES population. The initial value for 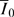 also closely matched the USDA estimated per capita food availability in 1974 adjusted for spoilage and wastage [10]. Our estimate of the food waste was given by:
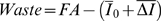
(14)where *FA* is the per capita food energy availability as estimated from US food balance sheets provided by the Food and Agriculture Organization [7]. To investigate how our calculation of food waste compares to current USDA estimates, we compared our estimated energy intake, 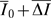, with the USDA per capita food availability corrected for spoilage and wastage.

To calculate the confidence intervals of our calculations, each model parameter value was randomly selected from a normal distribution with mean and standard deviations given in Table 1. The parameter ranges were estimated using the reported uncertainties of the measured parameter values, where available. In the case of the Forbes constant, *C*, and the physical activity parameter, *δ*, we chose a 50% uncertainty to reflect the potential for high variability of these parameters across the population. We performed 10^5^ simulations and report the mean and 95% confidence intervals for the predicted food intake and waste calculations.

**Table 1:** Mathematical model parameters.

*Parameter*	*Value (mean±SD)*	*Description*
*γ_FFM_*	22±4 kcal/kg/d	Resting metabolic rate coefficient for FFM
*γ_F_*	3.6±2 kcal/kg/d	Resting metabolic rate coefficient for F
*δ*	7±4 kcal/kg/d	Physical activity coefficient
*β*	0.24±0.1	Adaptive thermogenesis parameter
*η_FFM_*	230±100 kcal/kg	Energy cost for FFM deposition
*η_F_*	180±20 kcal/kg	Energy cost for F deposition
*C*	10.4±5 kg	Forbes body composition parameter
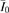	2100±100 kcal/d	Average energy intake in 1974
